# Effect of dexamethasone exposure on the neonatal unit on the school age lung function of children born very prematurely

**DOI:** 10.1371/journal.pone.0200243

**Published:** 2018-07-09

**Authors:** Christopher Harris, Siobhan Crichton, Sanja Zivanovic, Alan Lunt, Sandy Calvert, Neil Marlow, Janet L. Peacock, Anne Greenough

**Affiliations:** 1 MRC & Asthma UK Centre for Allergic Mechanisms in Asthma, King’s College London, London, United Kingdom; 2 Department of Women and Children’s Health, School of Life Course Sciences, Faculty of Life Sciences and Medicine, King's College London, London, United Kingdom; 3 School of Population Health and Environmental Sciences, King’s College London, London, United Kingdom; 4 Department of Child Health, St George's Hospital, London, United Kingdom; 5 Neonatal Medicine, University College, London, United Kingdom; 6 NIHR Biomedical Centre at Guy’s and St Thomas NHS Foundation Trust and King’s College London, London, United Kingdom; Centre Hospitalier Universitaire Vaudois, FRANCE

## Abstract

The objective of this study was to determine the impact of postnatal dexamethasone treatment on the neonatal unit on the school age lung function of very prematurely born children. Children born prior to 29 weeks of gestational age had been entered into a randomised trial of two methods of neonatal ventilation (United Kingdom Oscillation Study). They had comprehensive lung function measurements at 11 to 14 years of age. One hundred and seventy-nine children born at a mean gestational age of 26.9 (range 23–28) weeks were assessed at 11 to 14 years; 50 had received postnatal dexamethasone. Forced expiratory flow at 75% (FEF_75_), 50%, 25% and 25–75% of the expired vital capacity, forced expiratory volume in one second, peak expiratory flow and forced vital capacity and lung volumes including total lung capacity and residual volume were assessed. Lung function outcomes were compared between children who had and had not been exposed to dexamethasone after adjustment for neonatal factors using linear mixed effects regression. After adjustment for confounders all the mean spirometry results were between 0.38 and 0.87 standard deviations lower in those exposed to dexamethasone compared to the unexposed. For example, the mean FEF_75_ z-score was 0.53 lower (95% CI 0.21 to 0.85). The mean lung function was lower as the number of courses of dexamethasone increased. In conclusion, postnatal dexamethasone exposure was associated with lower mean lung function at school age in children born extremely prematurely. Our results suggest the larger the cumulative dose the greater the adverse effect on lung function at follow-up.

## Introduction

Corticosteroids administered to prematurely born infants can facilitate early extubation and reduce the rate of bronchopulmonary dysplasia (BPD) [1, 2]. Postnatally administered corticosteroids have, however, been shown to adversely affect lung growth in rats resulting in emphysematous lungs with fewer airspaces and delayed alveolarization [3]. There are limited and conflicting data regarding the long term pulmonary effects of postnatal corticosteroids on prematurely born children. In a study of 16 children born between 24 to 29 weeks of gestation, who had been entered into a randomised controlled trial (RCT) at 10 days of age, no significant differences were found in respiratory morbidity between 7.8 and 9.2 years [4]. In a follow up of another RCT, no statistically significant differences were found in lung function results (forced expiratory volume in one second (FEV_1_), forced vital capacity (FVC), FEV_1_/FVC ratio, peak expiratory flow (PEF), forced expiratory flow between 25% and 75% of VC (FEF_25-75%_)) at 13 to 17 years between the 68 prematurely born children who had received corticosteroids and the 74 who had received placebo at a median age of four weeks [5]. There were also no significant differences in inhaler use (relative risk (RR) 0.67, 95% CI 0.37 to 1.22), wheezing (RR 0.80, 95% CI 0.53 to 1.20) or cough (RR 1.19, 95% CI 0.67 to 2.23) between the two groups [5]. One study reported a positive effect in that only 48% of children who had received a 42 day tapering course of postnatal corticosteroids had an FEV_1_ below the normal range at 8 to 11 years of age in comparison to 68% of the controls (p = 0.03) [6]. The parent reported incidence of asthma, however, was similar in the two groups [6] and a further study of those children reported no significant differences in impaired aerobic fitness between the two groups [7]. In contrast, in a cross-sectional study, 105 prematurely born children who had been exposed to postnatal corticosteroids, had lower FEV_1_ (p = 0.01), FEF_25-75_% (p = 0.003), and PEF (p = 0.02) at age 9–11 years [8]. It is important to note that none of those study populations [5–8] had been exposed to postnatal surfactant and few to antenatal corticosteroids.

The United Kingdom Oscillation Study (UKOS) was a randomised trial of two modes of neonatal ventilation involving 797 infants born prior to 29 weeks of gestational age. More than 90% of the infants were exposed both to antenatal corticosteroids and postnatal surfactant [9]. We previously demonstrated that administration of postnatal dexamethasone was associated with significantly increased proportions of children with respiratory hospital readmission: (0.35 vs 0.15, difference = 0.20; 95% CI: 0.08 to 0.31) and neurodevelopmental impairment (0.59 vs 0.45, difference = 0.14; 95% CI: 0.02 to 0.26) after adjustment for confounding [10]. The aim of this present study was to analyse the lung function results of those entered into the UKOS trial at 11 to 14 years [11] to test the hypothesis that those exposed to postnatal dexamethasone had lower mean lung function results.

## Methods

Children from UKOS were invited to attend for follow-up at 11 to 14 years of age at King’s College Hospital NHS Foundation Trust. Ethical approval was granted by the South Thames Multicentre Research Ethics Committee and by the South West London National Research Ethics Service Committee for the follow up study [11]. Parents gave informed, written consent.

Lung function testing was carried out as previously described [11]. FEF_75_, FEF_50,_ FEF_25_ and FEF_25-75_, FEV_1_, PEF and FVC were assessed by spirometry. Lung volumes were assessed using a helium-dilution technique (functional residual capacity, FRC_He_) and plethysmography ((FRC_pleth_), total lung capacity (TLC) and residual volume (RV)). Respiratory resistance was measured by impulse oscillometry at 5Hz and 20Hz. The diffusing capacity of the lungs for carbon monoxide was measured using a single breath technique. All tests were done in accordance to guidance from the American Thoracic Society and the European Respiratory Society. The results were converted into z-scores to adjust for age, sex, and height [11], except for PEF and respiratory resistance which were expressed as the percentage predicted for height and sex [12, 13].

Data for postnatal corticosteroid exposure was captured when the original UKOS study was undertaken in 1998–2001. Correspondence confirmed that dexamethasone was the corticosteroid used in the postnatal period in all units. An average course of dexamethasone would be 0.25 mg twice daily for three days, followed by 0.15 mg twice daily for three days, followed by 0.05 mg twice daily for three days.

### Statistical analysis

To examine the associations between postnatal dexamethasone use and lung function at follow-up, linear mixed effects regression models were used to allow for clustering of data arising from multiple births [14]. All other predictor variables were forced into the model and treated as fixed effects. Measures of dexamethasone use considered were: a yes/no indicator, the number of courses of dexamethasone (none, one, two or three) and the length of exposure to dexamethasone (0, 1–6, 7–12 or >12 days) [10]. Initially associations were explored in univariable models and then in a multivariable model. To adjust for potential confounders, the following factors were included in the multivariable models: sex, birth weight z-score, gestational age, maternal smoking during pregnancy, oxygen dependency at 36 weeks postmenstrual age (PMA), major cranial ultrasound abnormality, air leak, patent ductus arteriosus (PDA), pulmonary haemorrhage, mode of ventilation and age at the time of follow-up assessment. Sensitivity analyses were conducted to also adjust for antenatal steroids and postnatal surfactant. Differences in mean lung function results by dexamethasone exposure were also shown as the difference in the proportions with abnormal lung function using the fifth centile in healthy children to define abnormal. The distributional approach was used for these calculations to retain the same power as the comparison of means [15, 16] and followed the approach we used in previous work [11]. (Table D in S1 File).

A further set of sensitivity analyses were conducted to explore the robustness of the adjustment for confounders. This used propensity score matching to compare outcomes in children who had and had not been exposed to dexamethasone. Full details are given in Table C in S1 File).

Subjects with complete data in the variables required for adjustment in the multivariable models or matching in the propensity score analysis were included in the analysis. Where one, but not all, lung function assessment results were available, cases were excluded only from analyses of the measures for which the data were missing. Analysis was conducted using Stata 13MP.

## Results

**Fig 1:**
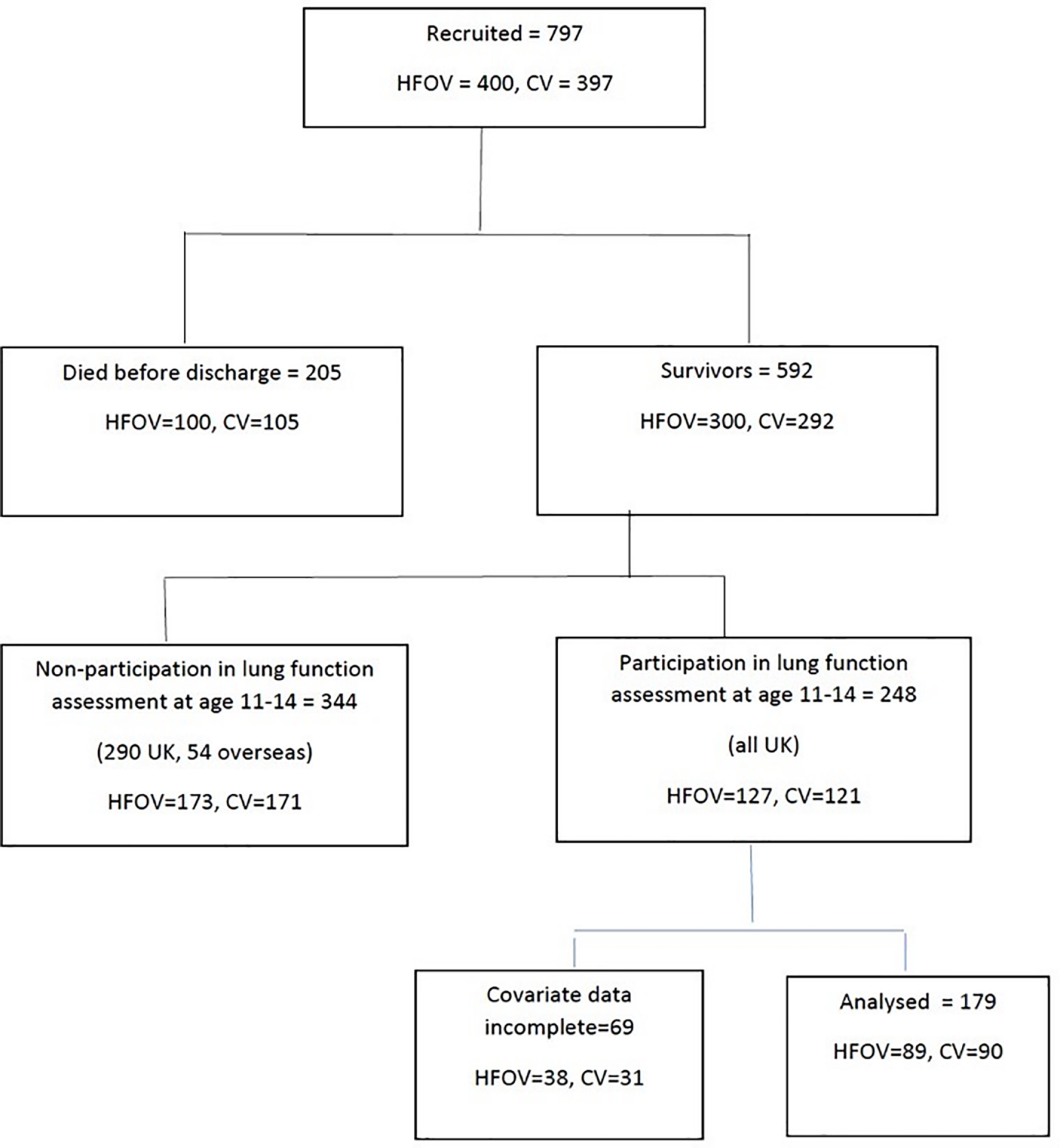
Study flow chart.

Two hundred and forty-eight children attended for lung function assessment; 179 had complete data at baseline and were included in these analyses (Fig 1). There were no significant differences between those with and without complete data for the majority of the lung function results, except for RV and FRC_pleth_ z scores (Table A in S1 File). Similarly, there were no significant differences in baseline characteristics comparing those included (n = 179) and those with incomplete data (n = 223) (Table B in S1 File).

Fifty children (28%) had received at least one course of dexamethasone in the neonatal period and 129 were not exposed to dexamethasone (Table 1). The children exposed to dexamethasone had significantly lower mean birth weight and gestational age, were less likely to be multiples, a greater proportion had an air leak and have lower Apgar five minute scores and a greater proportion were oxygen dependent at 36 weeks PMA (Table 1).

**Table 1:** Characteristics of children who were or were not exposed to dexamethasone. The data are presented as the mean (SD) or n (%) unless otherwise shown.

	No dexamethasone exposure	Dexamethasone exposure	p-value
N	129	50	
Birth weight (grams)	939 (205)	810 (175)	<0.001
Birthweight SDS	-0.63 (0.98)	-0.90 (1.12)	0.11
Gestational age (weeks)	26.7 (1.2)	25.6 (1.3)	<0.001
Male sex	60 (47%)	31 (62%)	0.063
Multiple birth	37 (29%)	7 (14%)	0.041
Oxygen dependency at 36 weeks PMA	62 (48%)	43 (86%)	<0.001
Major ultrasound abnormality in neonatal period	16 (12%)	7 (14%)	0.775
Airleak	9 (7%)	12 (24%)	0.001
Patent ductus arteriosus	34 (26%)	20 (40%)	0.074
Pulmonary haemorrhage	5 (3.9%)	5 (10%)	0.109
HFOV	60 (47%)	29 (58%)	0.168
Apgar score at 5 mins (mean, range)	9 (8–9)	8 (7–9)	<0.001
Maternal smoking in pregnancy	32 (25%)	9 (18%)	0.331
Antenatal steroids	14 (88%)	47 (96%)	0.160
Postnatal surfactant	127 (98%)	49 (98%)	>0.999

Comparison of the lung function results of those exposed and not exposed to dexamethasone demonstrated significant differences in favour of those unexposed in all the results using linear mixed model adjustment, with differences in the means of the spirometry results ranging from 0.38 to 0.87 standard deviations (Table 2). The differences in mean z-scores for the spirometry, measures are expressed as the equivalent percentage of children with abnormal function. For example, the percentage of children with an abnormal FEF_75_ is estimated as 21% in the non-exposed and 43% in the exposed group. The other spirometry measures show differences between the exposed and unexposed children ranging from 7.6 to 34 percentage points (Table 3).

**Table 2:** Lung function and postnatal dexamethasone exposure.

		No dexamethasone exposure	Dexamethasone exposure	Unadjusted		Adjusted using multiple regression	
Lung Function	N	Mean (SD)	Mean (SD)	Difference (95% CI)	p-value	Difference (95% CI)	p-value
FEF_75_ z score	179	-0.95 (0.91)	-1.45 (0.71)	-0.46 (0.74 to -0.18)	0.001	-0.53* (-0.85 to -0.21)	0.002
FEF_50_ z score	179	-1.04 (0.89)	-1.71 (0.81)	-0.66 (0.94 to -0.38)	<0.001	-0.74 (-1.05 to -0.43)	<0.001
FEF_25_ z score	179	-0.82 (0.91)	-1.53 (0.86)	-0.70 (-0.99 to -0.41)	<0.001	-0.75 (-1.07 to -0.44)	<0.001
FEF_25-75_ z score	169	-1.24 (1.07)	-1.98 (1.05)	-0.70 (-1.06 to -0.35)	<0.001	-0.70 (-1.08 to -0.33)	<0.001
FEV_1_ z score	179	-0.55 (1.03)	-1.44 (1.03)	-0.86 (-1.20 to—0.53)	<0.001	-0.87 (-1.24 to -0.51)	<0.001
FVC z score	179	-0.24 (0.96)	-0.73 (1.11)	-0.46 (-0.79 to -0.13)	0.006	-0.38 (-0.75 to -0.01)	0.043
FEV_1_:FVC z score	179	-1.17 (1.69)	-2.32 (2.11)	-1.14 (-1.74 to -0.55)	<0.001	-1.43 (-2.09 to -0.78)	<0.001
PEF % pred*	178	86.07 (14.64)	77.36 (13.98)	-8.34 (-13.06 to -3.62)	0.001	-10.74 (-16.06 to -5.41)	<0.001
RV z score	152	0.26 (1.09)	1.29 (1.67)	0.99 (0.53 to 1.45)	<0.001	0.86 (0.36 to 1.36)	0.001
FRC_pleth_ z	157	-0.11 (1.25)	0.39 (1.39)	0.49 (0.04 to 0.94)	0.031	0.39 (-0.11 to 0.90)	0.128
FRC_he_ z score	168	-0.73 (1.09)	-0.42 (1.00)	0.32 (-0.04 to 0.68)	0.083	0.27 (-0.13 to 0.66)	0.186
DL_CO_ z score	149	-0.93 (1.11)	-1.04 (1.02)	-0.06 (-0.46 to 0.34)	0.764	0.09 (-0.33 to 0.52)	0.658
At 5 Hz	170	96.06 (21.38)	100.11 (27.03)	4.60 (-3.22 to 12.42)	0.249	9.57 (1.13 to 18.02)	0.026
At 20 Hz	170	93.94 (19.82)	91.28 (4.46)	-2.28 (-10.13 to 5.58)	0.570	2.49 (-6.27 to 11.25)	0.578

*expressed as percentage predicted for height and sex

**Table 3:** Adjusted differences in mean lung function with equivalent differences in the proportions (as percentage) with abnormal lung function (below/above 5^th^ centile for healthy population*).

		Adjusted mean lung function (Table 2 in paper)	Percentage with abnormal lung function (rounded to 2 significant figures)		Adjusted difference in percentage with abnormal lung function
Lung Function measure	N	Difference (exposed-unexpo) (95% CI)	No dexamethasone exposure	Dexamethasone exposure	Difference (exposed-unexpo) (95% CI)
FEF_75_ z score+	179	-0.53 (-0.85 to -0.21)	21%	43%	22% (12 to 33%)
FEF_50_ z score	179	-0.74 (-1.05 to -0.43)	22%	57%	34% (24 to 44%)
FEF_25_ z score	179	-0.75 (-1.07 to -0.44)	15%	47%	31% (21 to 41%)
FEF_25-75_ z score	169	-0.70 (-1.08 to -0.33)	35%	64%	28% (18 to 39%)
FEV_1_ z score	179	-0.87 (-1.24 to -0.51)	13%	42%	29% (20 to 39%)
FVC z score	179	-0.38 (-0.75 to -0.01)	8%	15%	7.6% (1.7 to 14%)
FEV_1_:FVC z	179	-1.43 (-2.09 to -0.78)	38%	70%	32% (22 to 42%)
PEF % pred	178	-10.74 (-16.06 to -5.41)	-	-	-
RV z score	152	0.86 (0.36 to 1.36)	13%	35%	22% (12 to 32%)
FRC_pleth_ z	157	0.39 (-0.11 to 0.90)	-	-	-
FRC_he_ z score	168	0.27 (-0.13 to 0.66)	-	-	-
DL_CO_ z score	149	0.09 (-0.33 to 0.52)	-	-	-
At 5 Hz	170	9.57 (1.13 to 18.02)	-	-	-
At 20 Hz	170	2.49 (-6.27 to 11.25)	-	-	-

* Calculated where abnormality can be defined as below or above 5^**th**^ centile for a healthy population and using a distribution approach [15, 16]

+ The percentage of children with abnormal FEF_75_ was estimated as 21% in the non-exposed and 43% in the exposed group, a difference of 23% (95% CI: 13 to 33%) [12–13]

Sensitivity analyses adjusting for confounding using propensity score matching gave very similar results to the linear mixed models (Table D in S1 File). Further sensitivity analysis adjusting for antenatal steroids and postnatal surfactant made no appreciable difference (Table E in S1 File). Similarly, adjustment for tobacco smoke exposure at age 11–14 [17] did not affect the estimates or statistical significance (data not shown).

The mean lung function was lower in children who had received more courses of dexamethasone compared to one course and this remained significant after adjustment for most measures (Table 4). Similar trends were seen with the length of exposure with longer exposure being associated with poorer mean lung function which remained significant after adjustment (Table 5).

**Table 4:** Lung function and number of postnatal dexamethasone courses.

	Unadjusted					Adjusted				
	None	One course difference (95% CI) p-value	Two courses difference (95% CI) p-value	Three courses difference (95% CI) p-value	Overall p-value	None	One course difference (95% CI) p-value	Two courses difference (95% CI) p-value	Three courses difference (95% CI) p-value	Test for trend p-value
**N**	**129**	**35**	**11**	**4**		**129**	**35**	**11**	**4**	
FEF_75_ z score	ref	-0.37 (-0.68 to -0.06) 0.021	-0.59 (-1.11 to -0.08) 0.024	-0.92 (-1.76 to -0.08) 0.032	0.006	ref	-0.48 (-0.81 to -0.14) 0.006	-0.59 (-1.16 to -0.03) 0.040	-1.02 (-1.88 to -0.15) 0.021	0.001
FEF_50_ z score	ref	-0.52 (-0.84 to -0.21) 0.001	-1.01 (-1.53 to -0.49) <0.001	-0.95 (-1.80 to -0.10) 0.029	<0.001	ref	-0.65 (-0.98 to -0.33) <0.001	-0.99 (-1.53 to -0.44) <0.001	-1.15 (-1.99 to -0.31) 0.007	<0.001
FEF_25_ z score	ref	-0.61 (-0.94 to -0.28) <0.001	-0.85 (-1.39 to -0.30) 0.002	-1.13 (-2.01 to -0.24) 0.012	<0.001	ref	-0.71 (-1.05 to -0.38) <0.001	-0.78 (-1.34 to -0.22) 0.006	-1.22 (-2.08 to -0.37) 0.005	<0.001
FEF_25-75_ z score*	ref	-0.49 (-0.88 to -0.10) 0.014	-1.22 (-1.85 to -0.58) <0.001	1.19 (-2.23 to -0.15) 0.024	<0.001	ref	-0.60 (-1.00 to -0.20) 0.003	-0.96 (-1.62 to -0.31) 0.004	-1.29 (-2.31 to -0.28) 0.013	<0.001
FEV_1_ z score	ref	-0.64 (-1.02 to -0.27) 0.001	-1.32 (-1.93 to -0.70) <0.001	-1.60 (-2.60 to -0.60) 0.002	<0.001	ref	-0.74 (-1.13 to -0.36) <0.001	-1.16 (-1.80 to -0.52) <0.001	-1.65 (-2.63 to -0.67) 0.001	<0.001
FVC z score	ref	-0.26 (-0.63 to 0.10) 0.158	-0.82 (-1.43 to -0.22) 0.007	-1.19 (-2.18 to -0.21) 0.017	0.005	ref	-0.27 (-0.67 to 0.12) 0.174	-0.59 (-1.25 to 0.06) 0.077	-1.06 (-2.06 to -0.05) 0.039	0.009
FEV_1_:FVC z score	ref	-1.00 (-1.67 to -0.33) 0.003	-1.48 (-2.57 to -0.38) 0.008	-1.56 (-3.35 to 0.23) 0.088	0.002	ref	-1.30 (-2.00 to -0.59) <0.001	-1.82 (-2.99 to -0.64) 0.002	-1.98 (-3.77 to -0.18) 0.031	<0.001
PEF % of predicted**	ref	-8.11 (-13.35 to -2.87) 0.002	-3.51 (-12.10 to 5.07) 0.423	-23.27 (-37.33 to -9.22) <0.001	<0.001	ref	-10.27 (-15.85 to -4.70) <0.001	-6.55 (-15.88 to 2.77) 0.168	-26.51 (-40.91 to -12.11) <0.001	<0.001
RV z score***	ref	0.74 (0.23 to 1.25) 0.004	1.66 (0.81 to 2.52) <0.001	1.73 (-0.03 to 3.48) 0.053	<0.001	ref	0.68 (0.15 to 1.22) 0.011	1.47 (0.58 to 2.35) 0.001	1.27 (-0.45 to 2.99) 0.147	<0.0010.002
FRC_pleth_ z score****	ref	0.33 (-0.17 to 0.82) 0.194	1.10 (0.23 to 1.97) 0.013	0.45 (-1.34 to 2.24) 0.621	0.065	ref	0.25 (-0.29 to 0.78) 0.363	1.11 (0.18 to 2.04) 0.020	0.14 (-0.17 to 1.96) 0.877	0.063
FRC_he_ z score	ref	0.36 (-0.04 to 0.77) 0.080	0.11 (-0.54 to 0.76) 0.751	0.50 (-0.56 to 1.56) 0.353	0.299	ref	0.29 (-0.13 to 0.71) 0.178	0.18 (-0.52 to 0.88) 0.616	0.22 (-0.84 to 1.28) 0.689	0.338
DL_CO_ z score	ref	0.07 (-0.37 to 0.51) 0.762	-0.13 (-0.90 to 0.64) 0.735	-1.11 (-2.33 to 0.12) 0.077	0.332	ref	0.19 (-0.26 to 0.64) 0.412	0.11 (-0.68 to 0.89) 0.785	-1.00 (-2.21 to 0.21) 0.106	0.680
Respiratory resistance (% predicted)										
At 5 Hz	ref	4.03 (-4.65 to 12.72) 0.362	10.06 (-4.43 to 24.55) 0.174	-6.96 (-33.17 to 19.25) 0.603	0.436	ref	9.55 (0.60 to 18.50) 0.036	12.86 (-2.31 to 28.04) 0.097	-1.77 (-27.87 to 24.32) 0.894	0.093
At 20Hz	ref	0.82 (-7.91 to 9.56) 0.854	-5.54 (-20.16 to 9.07) 0.457	-25.71 (-51.84 to 0.41) 0.054	0.227	ref	4.63 (-4.63 to 13.89) 0.327	-0.59 (-16.24 to 15.06) 0.941	-20.74 (-47.49 to 6.02) 0.129	0.669

Footnote: differences are exposed–unexposed.

The children who received no dexamethasone are the reference group for all comparisons.

Some lung function results were only available for certain children

*169

**178

*** 152

**** 157

**Table 5:** Lung function and length of exposure to postnatal dexamethasone (days).

	Unadjusted					Adjusted				
	0	1–6, Difference (95% CI) p-value	7–12, Difference (95% CI) p-value	>12, Difference (95% CI) p-value	Overall p-value	0	1–6 days, Difference (95% CI) p-value	7–12 days, Difference (95% CI) p-value	>12 days, Difference (95% CI) p-value	Test for trend p-value
**N**	129	6	21	22		129	6	21	22	
FEF_75_ z score	ref	-0.23 (-0.89 to 0.43) 0.490	-0.34 (-0.74 to 0.05) 0.086	-0.63 (-1.01 to -0.25) 0.001	0.008	ref	-0.36 (-1.01 to 0.30) 0.286	-0.45 (-0.86 to -0.03) 0.034	-0.68 (-1.10 to -0.26) 0.002	<0.001
FEF_50_ z score	ref	-0.51 (-1.16 to 0.14) 0.126	-0.46 (-0.85 to -0.06) 0.025	-0.89 (-1.28 to -0.50) <0.001	<0.001	ref	-0.61 (-1.22 to 0.01) 0.054	-0.60 (-1.00 to -0.19) 0.004	-0.95 (-0.14 to -0.54) <0.001	<0.001
FEF_25_ z score	ref	-0.80 (-1.49 to -0.10) 0.025	-0.55 (-0.96 to -0.14) 0.009	-0.83 (-1.23 to -0.43) <0.001	<0.001	ref	-0.80 (-1.45 to -0.15) 0.017	-0.67 (-1.08 to -0.26) 0.001	-0.84 (-1.26 to -0.42) <0.001	<0.001
FEF_25-75_ z score*	ref	-0.67 (-1.46 to 0.13) 0.099	-0.55 (-1.04 to -0.06) 0.028	-0.84 (-1.33 to -0.35) 0.001	0.002	ref	-0.73 (-1.43 to -0.02) 0.043	-0.64 (-1.13 to -0.14) 0.001	-0.76 (-1.27 to -0.25) 0.003	0.008
FEV_1_ z score	ref	-0.87 (-1.77 to -0.07) 0.033	-0.53 (-1.00 to -0.06) 0.027	-1.17 (-1.63 to -0.71) <0.001	<0.001	ref	-0.88 (-1.63 to -0.14) 0.020	-0.63 (-1.10 to -0.16) 0.009	-1.13 (1.61 to -0.65) <0.001	<0.001
FVC z score	ref	-0.80 (-1.58 to -0.02) 0.045	-0.08 (-0.54 to -0.38) 0.731	-0.73 (-1.18 to -0.28) 0.001	0.005	ref	-0.78 (-1.55 to -0.01) 0.047	-0.07 (-0.55 to 0.40) 0.765	-0.57 (-1.06 to -0.09) 0.020	0.049
FEV_1_:FVC z score	ref	-0.45 (-1.84 to 0.94) 0.523	-0.98 (-1.81 to -0.15) 0.021	-1.51 (-2.32 to -0.69) <0.001	0.001	ref	-0.67 (-2.02 to 0.68) 0.329	-1.30 (-2.16 to -0.45) 0.003	-1.83 (-2.70 to -0.96) <0.001	<0.001
PEF % predicted**	ref	-8.99 (-19.85 to 1.87) 0.105	-9.55 (-16.21 to -2.59) 0.005	-7.61 (-14.10 to 1.13) 0.021	0.005	ref	-9.96 (-20.27 to 0.34) 0.058	-11.79 (-18.81 to -4.77) 0.001	-10.08 (-17.12 to -2.98) 0.005	<0.001
RV z score***	ref	1.58 (0.48 to2.67) 0.005	0.21 (-0.41 to 0.82) 0.510	1.59 (0.97 to 2.22) <0.001	<0.001	ref	1.37 (0.31 to 2.44) 0.012	0.21 (-0.41 to 0.82) 0.516	1.41 (0.77 to 2.05) <0.001	<0.001
FRC_pleth_ z score****	ref	0.78 (-0.25 to 1.80) 0.138	0.04 (-0.58 to 0.66) 0.899	0.83 (0.1 to 1.47) 0.011	0.040	ref	0.61 (-0.43 to 1.65) 0.250	-0.01 (-0.65 to 0.64) 0.980	0.77 (0.08 to 1.45) 0.028	0.083
FRC_he_ z score	ref	0.36 (-0.47 to 1.19) 0.392	0.28 (-0.22 to 0.77) 0.273	0.35 (-0.15 to 0.86) 0.170	0.380	ref	0.26 (-0.56 to 1.07) 0.538	0.22 (-0.29 to 0.73) 0.403	0.33 (-0.20 to 0.86) 0.223	0.184
DL_CO_ z score	ref	0.34 (-0.69 to 1.37) 0.517	-0.07 (-0.61 to 0.47) 0.790	-0.07 (-0.64 to 0.50) 0.803	0.902	ref	0.35 (-0.65 to 1.34) 0.493	0.07 (-0.48 to 0.62) 0.795	0.07 (-5.06 to 0.65) 0.803	0.7162
Respiratory resistance % predicted										
At 5 Hz	ref	17.35 (0.51 to 34.20) 0.043	-2.15 (-12.74 to 8.44) 0.690	8.85 (-2.33 to 20.03) 0.121	0.101	ref	20.41 (4.34 to 36.47) 0.013	3.30 (-7.56 to 14.16) 0.552	12.68 (0.90 to 24.47) 0.035	0.054
At 20 Hz	ref	11.41 (-6.57 to 29.39) 0.214	-4.36 (-15.06 to 6.35) 0.425	-4.35 (-15.73 to 7.03) 0.454	0.395	ref	12.24 (-5.30 to 29.79) 0.171	-0.05 (-11.33 to 11.23) 0.993	1.62 (-10.66 to 13.90) 0.796	0.828

Footnote: Diff: difference in means exposed—unexposed

The children who received no dexamethasone are the reference group for all comparisons

Some lung function results were only available for certain children

*169

**178

*** 152

**** 157

## Discussion

Our results from a secondary analysis suggest that postnatal dexamethasone exposure was associated with lower mean lung function at school age in children born very prematurely after adjusting for neonatal factors using careful adjustment for confounding with linear mixed effects modelling. In particular, we have shown significant differences in airway function and increased gas trapping as indicated by a higher residual volume. These results were replicated using a different form of adjustment, propensity score matching. The difference observed is substantial being over one half of a standard deviation for FEF_75_, equivalent to a difference of 22 percentage points in children with abnormal lung function. In addition, our results suggest that the longer the course or the greater the number of courses the greater the observed reduction in lung function. These results were consistent with our earlier study that showed an association between dexamethasone exposure and poor respiratory and neurological outcomes in infancy in the same population and using the same rigorous statistical approach to control for confounding [10]. In contrast, results of an earlier study had suggested that a longer duration of the corticosteroid might improve respiratory outcomes. Mean lung function at 15 years of age was significantly better in those who had received a 42 day course compared to those who had received an 18 day course [18]. There were, however no significant differences between those who received the 42 day course or the placebo and only 22 children in total were included in the study. Our analysis was of a larger cohort and we adjusted carefully for possible confounders.

Our results of impaired lung function following postnatal dexamethasone exposure are consistent with findings in animal models showing abnormal lung function. Although, we highlight that the significant reduction in airway function was more than the reduction in lung volume. In an early animal study [3], postnatal corticosteroids resulted in emphysematous lungs with fewer air spaces and delayed alveoarisation. A more recent study has highlighted that dexamethasone treatment of newborn rats resulted in delayed expression of elastin and smooth muscle actin and the parenchymal expression of tenascin-C (TNC) was delayed [19]. The authors postulated that neonatal corticosteroids impaired the first phase of alveolarisation by altering TNC and elastin expression [19]. In mice treated with dexamethasone, the mRNA expression level of fibrillin-1, which is a key protein reinforcing elastic fibres, were lower than in controls. Fibrillin-1 consists of microfibrils as a scaffold to form elastic fibres and fibulin-5. On the other hand, the peak mRNA expression of tropelastin, the main component in elastic fibres, occurred earlier in the dexamethasone group [20]. The authors postulated this imbalance in the expression of tropelastin and microfibril might interfere with the efficient formation of elastic fibres, resulting in thinning of the alveolar walls [20]. Indeed, in the dexamethasone group there were fewer and larger alveoli [20]. Furthermore, we did not find any significant difference in diffusing capacity results between the two groups.

Our study has strengths and limitations. We report a wide range of lung function measures at 11 to 14 years of age in large number of extremely prematurely born children. Ninety per cent of the children had been exposed to antenatal corticosteroids and nearly all received postnatal surfactant and hence our population is similar to those currently receiving intensive care. We included in our modelling oxygen dependency at 36 weeks PMA as this has been associated with poorer lung function at follow up. We do not report the results of a randomised trial, but analysed our data using rigorous statistical modelling to control for confounding due to differences in neonatal factors and clustering due to multiple births (25% in our sample). The analyses for dexamethasone exposure as a yes/no outcome were also undertaken using propensity score matching. It was not possible to use propensity score matching for the three measures of dexamethasone exposure, that is timing of administration, number of courses and days of exposure due to the small numbers in the different dexamethasone-use categories and so no sensitivity analyses were possible for those outcomes. We do not report whether the lung function abnormalities were associated with an increased need for medications, excess symptoms or exercise intolerance. We intend to investigate this when we assess the UKOS population when they are older.

There were no significant differences in the acute outcomes in the children who had been entered into our RCT of two neonatal ventilation modes [9], but at 11–14 years mean lung function results were superior in the HFOV group [11]. Adjustment for mode of ventilation was made in this present analysis and did not explain the difference in outcomes by dexamethasone exposure. We found no evidence of differences in those with complete or incomplete data and so we consider that our results are generalizable to extremely prematurely born children. These data are observational and so we cannot exclude the possibility that the associations observed are due to unmeasured residual confounding, but we made every effort to control for confounders.

In conclusion, our results suggest postnatal dexamethasone exposure was associated with lower lung function at 11–14 years of age in children born extremely prematurely. In addition, our results suggest the larger the cumulative dose the worse the lung function at follow-up.

## Supporting information

S1 File Online supplement. Table A: Lung function at follow up of children with and without complete data.
Table B: Baseline characteristics of the infants included and not included due to missing lung function data.
Table C: Mean FEF_75_ z-score by neonatal factors (n = 179).
Table D: Lung function and postnatal dexamethasone exposure: sensitivity analyses adjusted for confounding using propensity score matching.
Table E: Sensitivity analyses adjusting for antenatal steroids and postnatal surfactant.
Table F: Random effects estimates from adjusted models presented in Table 2 main paper.
(DOC)
