# Supplementary material for: Effect of dexamethasone exposure on the neonatal unit on the school age lung function of children born very prematurely
Source: PLoS One. 2018 Jul 9;13(7):e0200243. doi: 10.1371/journal.pone.0200243 (PMC6037362; doi:10.1371/journal.pone.0200243)
Supplement: S1 File — Table A: Lung function at follow up of children with and without complete data. Table B: Baseline characteristics of the infants included and not included due to missing lung function data. Table C: Mean FEF75 z-score by neonatal factors (n = 179). Table D: Lung function and postnatal dexamethasone exposure: sensitivity analyses adjusted for confounding using propensity score matching. Table E: Sensitivity analyses adjusting for antenatal steroids and postnatal surfactant. Table F: Random effects estimates from adjusted models presented in Table 2 main paper. (DOC) [file pone.0200243.s001.doc]

**S1 File: Supplement**

**Effect of dexamethasone exposure on the neonatal unit on adolescent lung function**

Christopher Harris, Siobhan Crichton, Sanja Zivanovic, Alan Lunt, Sandy Calvert, Neil Marlow, Janet L Peacock, Anne Greenough

**List of tables in S1 file: supplement**

Table A: Lung function at follow up of children with and without complete data.

Table B: Baseline characteristics of the infants included and not included due to missing lung function data

Table C: Mean FEF_75_ z-score by neonatal factors (n=179)

Table D: Lung function and postnatal dexamethasone exposure: sensitivity analyses adjusted for confounding using propensity score matching

Table E: Sensitivity analyses adjusting for antenatal steroids and postnatal surfactant

# Table F: Random effects estimates from adjusted models presented in table 2 main paper

# Further Baseline data (S1 file: Online supplement. Tables A-C)

# Table A shows lung function at follow up in children with or without complete data and shows little evidence of bias in our dataset.

# Table B shows reasonable balance according to whether follow-up lung function data were or were not available.

# Table C gives mean FEF_75_ z-score by neonatal factors and shows that most neonatal factors had a limited effect on later lung function. This supports our suggestion that the observed effects of dexamethasone are unlikely to be fully explained by uncontrolled confounding.

S1 File: Online supplement. Table A: Lung function at follow up of children with and without complete data.

|  |  | **Complete covariate data** | **Missing covariate data** |  |
| --- | --- | --- | --- | --- |
| **Lung Function** | **N** | **Mean(SD)**  **(n=179)** | **Mean(SD)**  **(n=69)** | **p-value** |
| FEF_75_ z score | 248 | -1.09 (0.89) | -1.03 (0.87) | 0.748 |
| FEF_50_ z score | 248 | -1.22 (0.92) | -1.20 (0.85) | 0.837 |
| FEF_25_ z score | 248 | -1.02 (0.95) | -0.95 (0.89) | 0.403 |
| FEF_25-75_ z score | 231 | -1.46 (1.11) | -1.46 (0.97) | 0.995 |
| FEV_1_ z score | 248 | -0.80 (1.10) | -0.71 (0.95) | 0.563 |
| FVC z score | 248 | -0.38 (1.02) | -0.32 (0.84) | 0.769 |
| FEV_1_:FVC z score | 248 | -1.49 (1.88) | -1.33 (1.50) | 0.527 |
| PEF percentage predicted | 247 | 83.6 (14.9) | 82.9 (16.3) | 0.991 |
| RV z score | 211 | 0.54 (1.35) | 0.06 (0.96) | 0.018 |
| FRC_pleth_ z score | 218 | 0.03 (1.31) | -0.40 (1.11) | 0.034 |
| FRC_he_ z score | 229 | -0.64 (1.07) | -0.77 (1.06) | 0.469 |
| DL_CO_ z score | 210 | -0.94 (1.09) | -0.97 (1.03) | 0.941 |
| Respiratory resistance -percentagepredicted |  |  |  |  |
| At 5 Hz | 237 | 97.2 (23.0) | 91.3 (19.6) | 0.098 |
| At 20 Hz | 237 | 93.2 (23.0) | 89.76 (22.5) | 0.380 |

S1 file: Online supplement. Table B: Baseline characteristics of the infants included and not included due to missing lung function data

|  | **Sample analysed with complete data N=179** | **Sample not analysed with incomplete data N=223** | **Comparison of complete and incomplete samples** |
| --- | --- | --- | --- |
| **Characteristics** | **% (n) or mean (SD)** | **% (n) or mean (SD)** | **p-value** |
| Birth weight | 882 (208) | 912 (211) | 0.15 |
| Gestational age | 26.5 (1.3) | 26.2 (1.4) | 0.06 |
| Male sex | 51% (91) | 54% (125) | 0.57 |
| Multiple birth | 25% (44) | 22% (52) | 0.59 |
| Postnatal dexamethasone use | 28% (50) | 30% (70) | 0.64 |
| Oxygen dependency at 28 days | 82% (147) | 81% (188) | 0.71 |
| Oxygen dependency at 36 weeks PMA | 59% (105) | 58% (135) | 0.88 |
| Oxygen dependency at hospital discharge | 24% (43) | 21% (50) | 0.50 |
| Major ultrasound abnormality in neonatal period | 13% (23) | 15% (36) | 0.46 |
| Maternal smoking in pregnancy | 23% (41) | 27% (58) | 0.31 |

S1 File: Online supplement. Table C: Mean FEF_75_ z-score by neonatal factors (n=179)

|  | **Mean FEF_75_ z-score (SD)** | **p-value** |
| --- | --- | --- |
| **Birth weight**  <860g (89)  ≥860g (90) | -1.12 (0.95)  -1.07 (0.83) | 0.71 |
| **Birthweight standard devaion score**  <-0.5 (90)  ≥-0.5 (89) | -1.16 (0.86)  -1.02 (0.91) | 0.28 |
| **Gestational age**  23-25wk (42)  26-28wk (137) | -1.11 (0.95)  -1.09 (0.87) | 0.91 |
| **Sex**  Girl (88)  Boy (91) | -1.00 (1.03)  -1.18 (0.71) | 0.18 |
| **Multiple birth**  Singleton (135)  Multiple (44) | -1.22 (0.82)  -0.70 (0.98) | <0.001 |
| **Oxygen dependency at 36 weeks PMA**  No (74)  Yes (105) | -0.95 (0.84)  -1.19 (0.91) | 0.08 |
| **Neonatal cranial ultrasound**  Normal (156)  Abnormal (23) | -1.09 (0.91)  -1.11 (0.72) | 0.92 |
| **Airleak**  No (158)  Yes (21) | -1.10 (0.89)  -1.02 (0.87) | 0.68 |
| **Patent ductus arteriosus**  No (125)  Yes (54) | -1.11 (0.92)  -1.05 (0.80) | 0.72 |
| **Pulmonary haemorrhage**  No (169)  Yes (10) | -1.07 (0.89)  -1.48 (0.85) | 0.15 |
| **Mode of ventilation**  CV (90)  HFOV (89) | -1.19 (0.79)  -0.99 (0.96) | 0.13 |
| **Apgar score at 5 mins**  <9 (82)  ≥9 (97) | -1.06 (0.95)  -1.12 (0.83) | 0.66 |
| **Maternal smoking in pregnancy**  No (138)  Yes (41) | -1.01 (0.86)  -1.04 (0.99) | 0.65 |
| **Antenatal steroids**  No (17)  Yes (161) | -1.09 (0.67)  -1.09 (0.91) | 0.98 |

# Propensity Score Matching (S1 File: Online supplement. Table D)

# Since the use of postnatal dexamethasone is so highly confounded by neonatal factors, we also used propensity score matching for dexamethasone exposure (yes/no) [S4] as an alternative way of adjustment in addition to linear mixed model regression. Propensity score (PS) matching works differently to multiple regression in that it matches the subjects as closely as possible using baseline factors prior to analysis so that the study closely resembles a randomised trial

# The matching algorithm used was up to three nearest neighbours for each case. This approach was used because it leads to less bias than if the single nearest neighbour approach is used without replacement [S4]. Logistic regression models were used to assign a probability of steroid use to each child based on their baseline characteristics. The model included data collected prior to the initiation of steroid use, namely: sex, birth weight, birth weight z-score, gestational age in weeks (in keeping with the original trial’s randomisation strata), smoking in pregnancy, multiple birth, ventilation group and Apgar score at five minutes. Children who received dexamethasone, were then matched to three children who did not receive dexamethasone with the closest propensity scores.

# The main challenge of the PS method is to obtain close matches for all subjects. Inspection of the table of variables by groups before and after matching showed substantial improvement achieved by PS with no significant imbalance for any variable. It was not possible to use propensity score matching for three measures of dexamethasone exposure, that is timing of administration, number of courses and days of exposure due to the small numbers in the different dexamethasone-use categories. For this reason, only adjustment by multivariable logistic regression was undertaken for those measures. A further limitation of PS matching in this context is that it is difficult to adjust for clustering within propensity score models.

S1 File: Online supplement. Table D: Lung function and postnatal dexamethasone exposure: sensitivity analyses adjusted for confounding using propensity score matching

|  |  | **No dexamethasone exposure** | **Dexamethasone exposure** | **Adjusted using multiple regression (main analysis)** | | **Adjusted using propensity score matching (sensitivity)** | |
| --- | --- | --- | --- | --- | --- | --- | --- |
| **Lung Function** | **N** | **Mean**  **(SD)** | **Mean**  **(SD)** | **Difference**  **(95% CI)** | **p-value** | **Difference**  **(95% CI)** | **p-value** |
| FEF_75_ z score | 179 | -0.95  (0.91) | -1.45  (0.71) | -0.53*  (-0.85 to -0.21) | 0.002 | -0.51  (-0.89 to -0.13) | 0.009 |
| FEF_50_ z score | 179 | -1.04  (0.89) | -1.71  (0.81) | -0.74  (-1.05 to -0.43) | <0.001 | -0.54  (-0.93 to -0.14) | 0.006 |
| FEF_25_ z score | 179 | -0.82  (0.91) | -1.53  (0.86) | -0.75  (-1.07 to -0.44) | <0.001 | -0.51  (-0.78 to -0.24) | <0.001 |
| FEF_25-75_ z score | 169 | -1.24  (1.07) | -1.98  (1.05) | -0.70  (-1.08 to -0.33) | <0.001 | -0.55  (-0.99 to -0.11) | 0.014 |
| FEV_1_ z score | 179 | -0.55  (1.03) | -1.44  (1.03) | -0.87  (-1.24 to -0.51) | <0.001 | -0.62  (-1.00 to -0.24) | 0.002 |
| FVC z score | 179 | -0.24  (0.96) | -0.73  (1.11) | -0.38  (-0.75 to -0.01) | 0.043 | -0.23  (-0.59 to 0.14) | 0.221 |
| FEV_1_:FVC z | 179 | -1.17  (1.69) | -2.32  (2.11) | -1.43  (-2.09 to -0.78) | <0.001 | -1.16  (-1.98 to -0.34) | 0.006 |
| PEF % pred* | 178 | 86.07  (14.64) | 77.36  (13.98) | -10.74  (-16.06 to -5.41) | <0.001 | -7.42  (-11.5 to -3.4) | <0.001 |
| RV z score | 152 | 0.26  (1.09) | 1.29  (1.67) | 0.86  (0.36 to 1.36) | 0.001 | 0.67  (0.27 to 1.07) | 0.001 |
| FRC_pleth_ z | 157 | -0.11  (1.25) | 0.39  (1.39) | 0.39  (-0.11 to 0.90) | 0.128 | 0.37  (0.01 to 0.73) | 0.042 |
| FRC_he_ z score | 168 | -0.73  (1.09) | -0.42  (1.00) | 0.27  (-0.13 to 0.66) | 0.186 | 0.30  (-0.06 to 0.67) | 0.106 |
| DL_CO_ z score | 149 | -0.93  (1.11) | -1.04  (1.02) | 0.09  (-0.33 to 0.52) | 0.658 | -0.01  (-0.61 to 0.59) | 0.968 |
| At 5 Hz | 170 | 96.06  (21.38) | 100.11 (27.03) | 9.57  (1.13 to 18.02) | 0.026 | 1.22  (-5.42 to 7.86) | 0.719 |
| At 20 Hz | 170 | 93.94  (19.82) | 91.28  (4.46) | 2.49  (-6.27 to 11.25) | 0.578 | -1.57  (-8.79 to 5.64) | 0.669 |

# Further details on statistical analysis (S1 File: Online supplement. Tables E and F)

# Since differences in mean z-scores can be difficult to interpret, we have additionally presented the equivalent difference in the proportion of children with abnormal lung function. To do this we have used the 5^th^ centile for normal to define the cut-off between ‘normal’ and ‘abnormal’. Since in healthy children the z-score has a Normal (Gaussian) distribution, the cut-point for abnormality is defined as z< -1.645, the 5^th^ centile of the Normal Distribution.

# The proportion abnormal is not calculated using the data values themselves but using a statistical model to gain precision. These calculations are similar to those performed to calculate reference ranges. The calculations used a statistical method called the ‘distributional approach’[table 3] The distributional approach provides more precise values than we would obtain had we used the data alone. In the present study, the adjusted estimates from the multivariable mixed model analyses were used to estimate the difference in the proportion of children who have abnormal lung function in those children who were and were not exposed to steroids. The calculations use the same adjusting factors to allow for confounding neonatal factors as the main analyses [table 3].

# The differences for all analyses in main paper Table 2 are shown in Table 3. They show that what may seem to be quite small differences in mean z-score, eg, 0.53 standard deviations for FEF_75_, translate into quite substantial differences in the percentage that have abnormal lung function results, 22 percentage points, and so the additional data help to make the results more clinically meaningful.

# Table S5 is a further sensitivity analysis that adjusts for antenatal steroids and postnatal surfactant. This table shows no appreciable effect of this change in the modelling. Table S6 gives the random effects estimates for the mixed effects models shown in the main paper, table 2.

# S1 File: Online supplement. Table E: Sensitivity analyses adjusting for antenatal steroids and postnatal surfactant

|  |  | **No dexamethasone exposure** | **Dexamethasone exposure** | **Adjusted using original variables (Table 2 in text)** | **Adjusted using original variables + antenatal steroids, postnatal surfactant** |
| --- | --- | --- | --- | --- | --- |
| **Lung Function** | **N** | **Mean**  **(SD)** | **Mean**  **(SD)** | **Difference (exposed-unexpo) (95% CI)** | **Difference (exposed-unexpo)**  **(95% CI)** |
| FEF_75_ z score | 179 | -0.95  (0.91) | -1.45  (0.71) | -0.53  (-0.85 to -0.21) | -0.52  (-0.84 to -0.20) |
| FEF_50_ z score | 179 | -1.04  (0.89) | -1.71  (0.81) | -0.74  (-1.05 to -0.43) | -0.73  (-1.04 to -0.43) |
| FEF_25_ z score | 179 | -0.82  (0.91) | -1.53  (0.86) | -0.75  (-1.07 to -0.44) | -0.77  (-1.09 to -0.46) |
| FEF_25-75_ z score | 169 | -1.24  (1.07) | -1.98  (1.05) | -0.70  (-1.08 to -0.33) | -0.68  (-1.06 to -0.31) |
| FEV_1_ z score | 179 | -0.55  (1.03) | -1.44  (1.03) | -0.87  (-1.24 to -0.51) | -0.88  (-1.24 to -0.51) |
| FVC z score | 179 | -0.24  (0.96) | -0.73  (1.11) | -0.38  (-0.75 to -0.01) | -0.40  (-0.77 to -0.02) |
| FEV_1_:FVC z | 179 | -1.17  (1.69) | -2.32  (2.11) | -1.43  (-2.09 to -0.78) | -1.42  (-2.08 to -0.75) |
| PEF % pred* | 178 | 86.07  (14.64) | 77.36  (13.98) | -10.74  (-16.06 to -5.41) | -10.75  (-16.15 to -5.35) |
| RV z score | 152 | 0.26  (1.09) | 1.29  (1.67) | 0.86  (0.36 to 1.36) | 0.86  (0.38 to 1.39) |
| FRC_pleth_ z | 157 | -0.11  (1.25) | 0.39  (1.39) | 0.39  (-0.11 to 0.90) | 0.40  (-0.12 to 0.91) |
| FRC_he_ z score | 168 | -0.73  (1.09) | -0.42  (1.00) | 0.27  (-0.13 to 0.66) | 0.21  (-0.18 to 0.61) |
| DL_CO_ z score | 149 | -0.93  (1.11) | -1.04  (1.02) | 0.09  (-0.33 to 0.52) | 0.06  (-0.36 to 0.49) |
| At 5 Hz | 170 | 96.06  (21.38) | 100.11  (27.03) | 9.57  (1.13 to 18.02) | 8.60  (0.10 to 17.10) |
| At 20 Hz | 170 | 93.94  (19.82) | 91.28  (4.46) | 2.49  (-6.27 to 11.25) | 1.84  (-7.02 to 10.71) |

# S1 File: Online supplement. Table F: Random effects estimates from adjusted models presented in table 2 main paper

| **Lung Function** | **SD Intercept** | **SD Residual** |
| --- | --- | --- |
| FEF_75_ z score | 0.54 | 0.62 |
| FEF_50_ z score | 0.60 | 0.54 |
| FEF_25_ z score | 0.52 | 0.63 |
| FEF_25-75_ z score | 0.80 | 0.55 |
| FEV_1_ z score | 0.58 | 0.74 |
| FVC z score | 0.58 | 0.76 |
| FEV_1_:FVC z score | 1.25 | 1.19 |
| PEF % pred* | 10.96 | 8.55 |
| RV z score | 0.70 | 0.93 |
| FRC_pleth_ z | 0.80 | 0.97 |
| FRC_he_ z score | 0.62 | 0.78 |
| DL_CO_ z score | 0.73 | 0.70 |
| At 5 Hz | 16.41 | 14.14 |
| At 20 Hz | 15.42 | 16.13 |

# References

1. Zivanovic S, Peacock J, Alcazar-Paris M, Lo J, Lunt A, Marlow N, et al; United Kingdom Oscillation Study Group. Late outcomes of a randomized trial of high-frequency oscillation in neonates. N Engl J Med. 2014;370: 1121-1130.
2. Caliendo and Kopeinig 2003 (<http://ftp.iza.org/dp1588.pdf>) (web document).
3. Peacock JL, Sauzet O, Ewings SM, Kerry SM. Dichotomizing continuous data while retaining statistical power using a distributional approach. Stat Med. 2012;31: 3089–3103.
4. Sauzet O, Breckenkamp J, Borde T, Brenne S, David M, Razum O, et al. A distributional approach to obtain adjusted comparisons of proportions of a population at risk. Emerg Themes Epidemiol. 2016;13: 8.
